# Therapy strategies of fifth metatarsal base fracture with lateral collateral ligament injury

**DOI:** 10.1186/s13018-022-02935-7

**Published:** 2022-01-24

**Authors:** Hongbin Cao, Nan Li, Guixin Wang, Jinquan He

**Affiliations:** The First Department of Foot and Ankle Surgery, Tianjin Hospital, Tianjin, 300211 China

**Keywords:** Fifth metatarsal base fracture, Ankle, Lateral collateral ankle ligament

## Abstract

**Background:**

Fifth metatarsal base fracture (fifth MBF) and lateral collateral ankle ligament (LCAL) injury are mainly caused by plantar flexion and inversion of the foot. However, there is no relevant report on the incidence, injury type and treatment principle of the fifth MBF combined with an LCAL injury.

**Materials and methods:**

We retrospectively analyzed 61 patients with fifth MBF. After admission, patients were given the symptomatic treatment and underwent standard anteroposterior (AP), 30-degree oblique foot radiographs, ankle MR and/or ultrasonic examination. The type of surgery varied base on the individual patients (type of fracture with/without lateral collateral ankle ligament injury).

**Results:**

In 61 patients, there were 39 patients with LCAL injury. Among the 39 patients with LCAL injury, 24 patients with Grade I–II injury, 6 patients with Grade III injury, and 9 patients with avulsion fractures. There was no significant difference between the patients without LCAL injury and the patients with LCAL injury in terms of age (*p* = 0.67) and gender (*p* = 0.575). The incidence of fifth MBF with LCAL injury accounted for 63.93% of fifth metatarsal base fracture; the most common causes of injury included sprains and falls. The average fracture healing time was 8.3 (range, 6–12) weeks. For fifth MBF with displaced more than 2 mm, hook plate or lag screw was used for fixation; for complete rupture of LCAL, suture anchor was used to repairing the ligament; for partial LCAL injury, plaster was used for fixation after surgery; for avulsion fractures, cannulated screw or suture anchor was used for repair. None of the patients had complications such as delayed union, nonunion, and incision infection.

**Conclusion:**

Early diagnosis and appropriate treatment can obtain good therapeutic results in fifth MBF patients combined with LCAL injury. Moreover, defining a treatment plan for ligament injury is essential for reducing postoperative complications. This study provides a basis for epidemiology, diagnosis, and treatment of fifth MBF with LCAL injury.

## Introduction

Fifth metatarsal fracture is one of the most common foot injuries occurring due to trauma or repetitive microstress, with an incidence of approximately 56–68% [1–3], where approximately 70% of patients present with proximal fractures. According to Lawrence–Botte classification, fractures of the proximal fifth metatarsal are divided into zone I (the most common one, tuberosity avulsion fractures), zone II (Jones' fracture), and zone III (proximal shaft stress fractures) [4, 5]. Most fifth metatarsal base fractures are low-energy injuries caused by plantar flexion and inversion of the foot [6]. Yet, fifth metatarsal base fractures may be combined with the lateral collateral ankle ligament (LCAL), which is composed of the anterior talofibular ligament (ATFL), calcaneofibular ligament (CFL), and posterior talofibular ligament (PTFL). In case LCAL injury without properly diagnosed, it may lead to chronic ankle instability, talar cartilage injury, and ankle traumatic arthritis [7–9]. Thus, the accurate diagnosis of lateral collateral ankle ligament in patients with a fifth metatarsal fracture is extremely important.

At present, there is a lack of studies on the incidence, injury types, and treatment methods of fifth MBF with an LCAL injury. In this study, we explored the incidence of fifth MBF with LCAL injury so as to explore the injury types and treatment principles.

## Materials and methods

### Patients

This retrospective study analyzed 61 patients (61 feet) with fifth MBF treated in our department from January 2017 to June 2019. There were 40 male patients and 21 female patients; their average age was 44 (range, 20–69) years; there were 34 patients with injuries on the left side and 27 patients with injuries on the right side; there were 28 sprains, 25 falls, 6 car accidents, and 2 slips and falls. Inclusion criteria were given as follows: patients older than 18 years with a fresh fifth metatarsal base fracture. All the patients have gone through standard standing anteroposterior (AP), 30-degree oblique foot radiographs, ankle MR and/or ultrasonic examination.

Exclusion criteria were given as follows: patients with fracture of other parts of the foot, orold ankle ligament injury, or open fracture, or nerve and blood vessel injury.

### Post-admission treatment

After admission, patients were given the symptomatic treatment of the elevation of the affected limb and the relief of swelling and pain. They also underwent AP, oblique foot radiographs, ankle MR and/or ultrasonic examination. The average time from injury to surgery was 3.6 (range, 2–8) days. Among 61 patients, there were 4 patients of fifth MBF with undisplacement; 39 patients with LCAL injury, including 24 patients with Grade I-II LCAL injuries, 3 patients with ATFL injury (rupture at lateral malleolus), 1 case with CFL injury (rupture at calcaneus), 2 patients with ATFL + CFL injuries (rupture at lateral malleolus); 8 patients with a lateral malleolus avulsion fracture and 1 case with talus avulsion fracture; 22 patients without LCAL injury. According to Lawrence classification, all fractures were classified as zone I fracture [5], and LCAL injuries were classified into Grade I injury: partial tear of a ligament; Grade II injury: incomplete tear of a ligament, with moderate functional impairment; Grade III injury: complete tear and loss of integrity of a ligament [10].

### Nonoperative treatment

4 patients with undisplaced (displace less than 2 mm) of the fifth MBF which showed no LCAL injury on ankle by MR and/or ultrasonic examination were used nonoperative treatment. The plaster or brace was used for fixation for 4 weeks, functional exercise and partial weight-bearing were taken after those 4 weeks, and normal life was restored after 3 months.

### Operative treatment

After successful epidural anesthesia, the patient was placed in the supine position and pressurized with a pneumatic tourniquet. Different surgeries were used for different types of fractures.

#### Fifth metatarsal base fracture

**Fig. 1:**
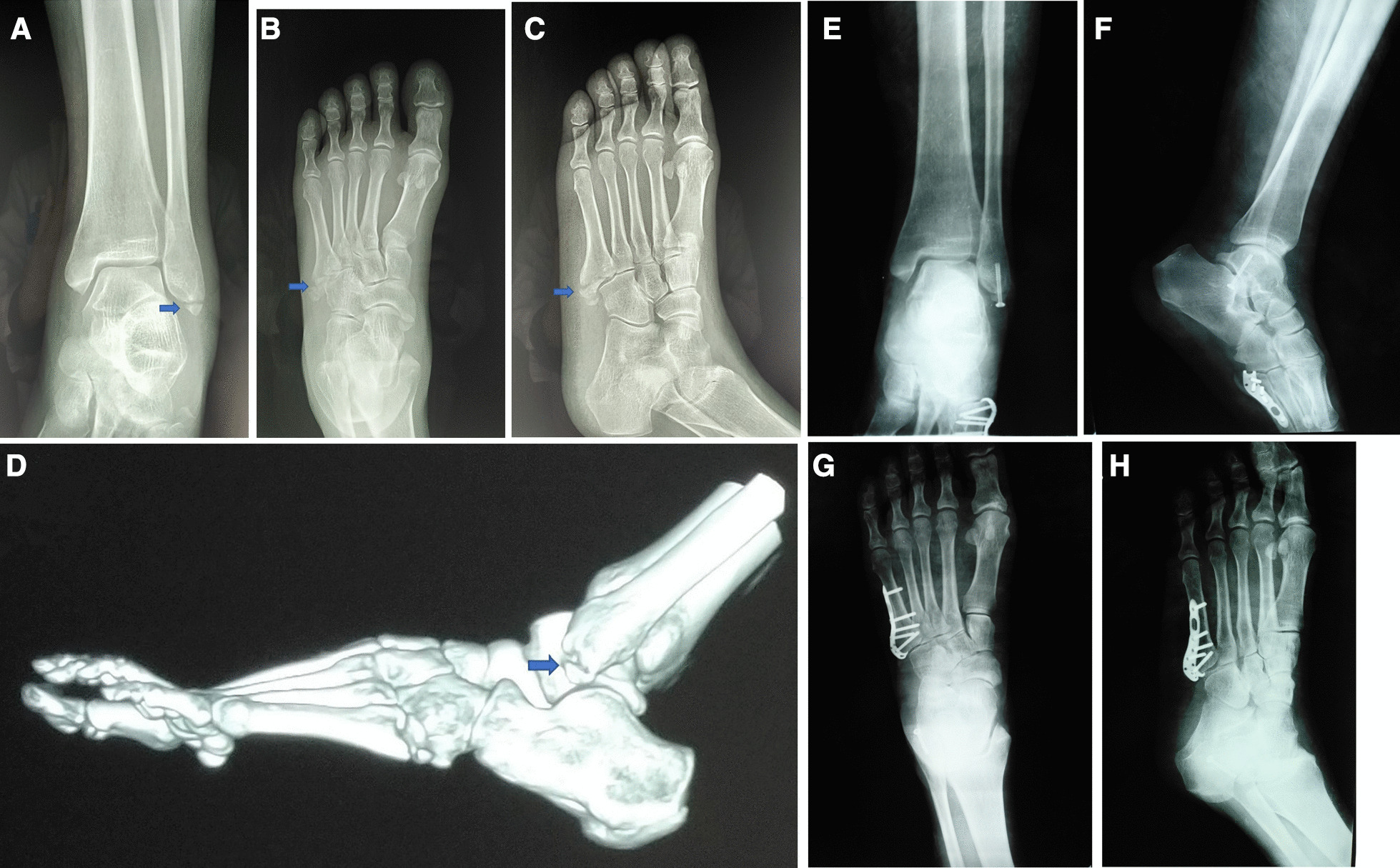
**A** Ankle anteroposterior /lateral radiographs showed avulsion fracture at the tip of lateral malleolus, arrow showed lateral malleolus fracture. **B**, **C** Preoperative foot anteroposterior/oblique radiographs showed displaced-fifth metatarsal base fracture, arrow showed fifth metatarsal base fracture. **D** Preoperative CT showed lateral malleolus avulsion fracture block and fifth metatarsal base fracture, arrow showed lateral malleolus fracture. **E**, **F** Postoperative ankle radiographs showed that lateral malleolus avulsion fracture was fixed with a cannulated screw. **G**, **H** Postoperative foot radiographs showed that the fifth metatarsal base fracture was fixed with a hook plate, and the fracture was anatomically reduced

For the fifth MBF with displacement more than 2 mm and involving more than 30% of the fifth metatarsal base-cuboid joint adopted operative intervention. A longitudinal skin incision was made plantar lateral to the fifth MBF, and the skin and subcutaneous soft tissue were cut in turn. Meanwhile, we paid special attention to protect the sural nerve. Then, the fracture site was exposed, and hematoma and soft tissue at the fracture end were removed to show the attachment point of the roneus brevis at the proximal fracture block. Afterward, towel clip were used to reduce fracture, the assistant kept the foot in abduction position, and "C" arm was used to confirm that the fracture site was aligned well, and the joint surface was flat. Eventually, a hook plate (Double Medical, Xiamen, The People's Republic of China) or lag screw was used to fix the fracture (Fig. 1).

#### Lateral malleolus avulsion fracture

A straight incision was made at the tip of the lateral malleolus, and the skin and subcutaneous tissue were cut in turn to expose the fracture end. Then towel forceps were used to reduce fracture, "C" arm was used to confirm the anatomical reduction of the fracture. Eventually, cannulated screw guide pins were driven in, and one to two cannulated screws (DePuy Synthes, West Chester, USA) were used for fixation (Fig. 1).

#### Ligament repair

Ligament repair was made for patients with complete ligament rupture (Grade III injury) showed by preoperative MRI and/or ultrasonic examination; the anterior drawer test and inversion test stress radiographs of the affected ankle side were performed under the "C" arm for preoperative MRI and/or ultrasonic examination showed Grade I–II injury and compared with the healthy side, a significant difference between the two sides was positive [11], ligament exploration was necessary for those patients. For patients with Grade I–II injury whose result of the anterior drawer and inversion stress radiographs was negative, the fifth MBF was treated with open reduction and internal fixation and plaster fixation after the surgery.

For patients with Grade III ligament injury and whose results of the anterior drawer and inversion stress radiographs was positive, a lateral malleolus arc incision was made, and the skin, subcutaneous tissue, and fascia were cut to expose anterior talofibular ligament (ATFL) and calcaneofibular ligament (CFL), the ligament was repaired with 3.0 mm suture anchor (Arthrex, Naples, USA).

**Fig. 2:**
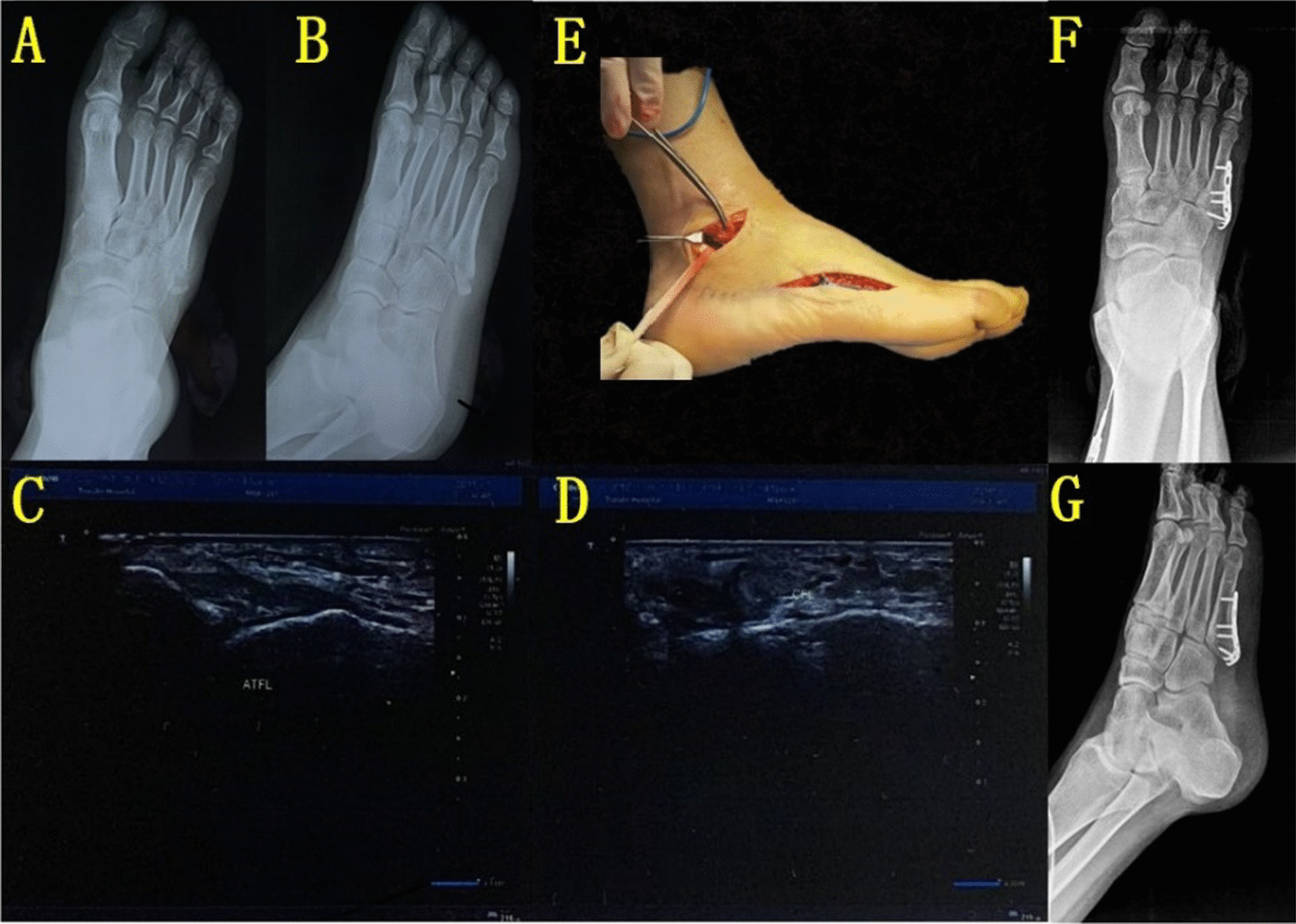
**A, B** Preoperative foot radiographs showed displaced-fifth metatarsal base fracture, arrow showed fifth metatarsal base fracture. **C**, **D** Preoperative ultrasonic examination showed partial injuries of anterior talofibular ligament and calcaneofibular ligament. **E** Intraoperative exploration showed calcaneofibular ligament completely rupture, but anterior talofibular ligament continuously exists with acceptable tension, arrow showed calcaneofibular ligament. **F**, **G** Postoperative foot radiographs showed that the fifth metatarsal base fracture was fixed with a hook plate, and the fracture was anatomically reduced

For patients with avulsion fractures whose fracture block was too small to be fixed, the fracture block was taken out during surgery. The ligament was repaired with a 3.0 mm suture anchor (Arthrex, Naples, USA), and the anterior drawer and inversion stress radiographs of the affected ankle side were examined again to confirm that the results of the anterior drawer test and inversion test were negative (Fig. 2).

### Postoperative treatment

No additional fixation was needed for patients without LCAL injury after the surgery. However, for those with LCAL injury, the short leg plaster or brace was used for fixation for 3 weeks. Briefly, toes were actively flexed and stretched, and the wound was sutured 14 days after the surgery. After 2 weeks, the plaster was removed, and the ankle dorsiflexion was practiced. After 6 weeks, the weight was partially loaded, and after 12 weeks, the weight was completely loaded.

### Assessment indicators

The fracture healing was assessed in line with the imaging examination during follow-up. X-ray film showed that the fracture line was blurred, and there was continuous callus passing through the fracture line, which was assessed as fracture healing.

When the internal fixation was taken out (at the last follow-up), the ankle function was assessed, and the ultrasonic or MRI ankle examination was carried out to observe the recovery of LCAL injury.

Ankle-Hindfoot Rating System developed by American Orthopaedic Foot and Ankle Society (AOFAS) was used to assess ankle function [12]. The rating includes pain (40 points), function (50 points), and alignment (10 points). The total score was 100 points, of which the 90–100 points indicated excellent results, 75–89 points good results, 50–74 points fair results, and < 50 poor results [12].

### Statistical analysis

SPSS software (Version 23.0, SPSS Inc, Chicago, IL, USA) was used for statistical analysis. T-test was used for counting data, which were expressed by mean ± standard deviation (X ± SD). The Chi-square test was used for classification data, which was expressed by the ratio (%). A *p* < 0.05 was considered statistically significant. G*Power (Version 3.1.9.6) was used for different statistical tests.

## Results

### Patient data

A total of 61 patients with fifth MBF were enrolled, including 22 patients without LCAL injury and 39 patients with LCAL injury. There was no significant difference between the patients without LCAL injury and the patients with LCAL injury in terms of age (*p* = 0.67, Table 1) and gender (*p* = 0.575, Table 1). There were 39 patients with LCAL injury, including 24 patients with partial ligament injury (Grade I, II injuries), 6 patients with ATFL and CFL injuries (Grade III injury), and 9 patients with avulsion fractures in different parts (8 patients with lateral malleolus avulsion fracture, and 1 talus avulsion fracture). Among the three types of injuries, the incidence of partial ligament injury was the highest (61.54%, Table 2), followed by avulsion fracture (23.08%, Table 2). Although there was a higher incidence of male patients with Grade III injury, there was no significant difference in gender and age statistics among the three groups.

**Table 1:** Descriptive data of 61 patients with fifth metatarsal base avulsion fracture

Injury type	Number of feet (%)	Gender (%)		Age (years)
		Male	Female	
Without LCAL injury	22 (36.07%)	13 (59.09%)	9 (40.91%)	45 ± 12.58
LCAL injury (Grade I–III injury and avulsion fracture)	39 (63.93%)	27 (69.23%)	12 (30.77%)	43.46 ± 14.22
*t* value				0.42
*p* value	–	0.575		0.67

LCAL, lateral collateral ankle ligament. Grade I injury, partial tear of a ligament. Grade II, incomplete tear of a ligament, with moderate functional impairment. Grade III, complete tear and loss of integrity of a ligament

**Table 2:** Descriptive data of 39 Patients with Fifth Metatarsal Base Fracture with LCAL Injury

Injury type	Number of feet (%)	Gender (%)		Age (years)
		Male	Female	
Partial LCAL injury (Grade I, II injury)^1^	24 (61.54%)	15 (62.5%)	9 (37.5%)	43.75 ± 15.4
Grade III injury^2^	6 (15.38%)	6 (100%)	0 (0%)	39.33 ± 12.06
With avulsion fracture^3^	9 (23.08%)	6 (66.67%)	3 (33.33%)	45.44 ± 13.06
*p* value	–	0.091^a^ 0.579^b^ 0.185^c^		0.52^a^ 0.38^b^ 0.77^c^

LCAL, lateral collateral ankle ligament. Grade I injury, partial tear of a ligament. Grade II, incomplete tear of a ligament, with moderate functional impairment. Grade III, complete tear and loss of integrity of a ligament

^a^*p* value between Partial LCAL injury and Grade III injury

^b^*p* value between Partial LCAL injury and with avulsion fracture

^c^*p* value between Grade III injury and with avulsion fracture

### Post-treatment assessment

The average follow-up time of all patients was 16.8 months (range, 13–24 months) (follow-up in the first 3 months, once a month; follow-up in the late once every 3 months). During the follow-up, we assessed surgery-related complications (limited ankle movement, traumatic arthritis, and lateral ankle instability), AOFAS Ankle-Hindfoot Rating, and fracture healing. No incision infection, loss of fracture reduction or nonunion occurred after the surgery in patients with various types of injuries during the follow-up. The average time of fracture healing was 8.3 (range, 6–12) weeks. At the last follow-up, AOFAS Ankle-Hindfoot Rating was carried out, and the ratings of patients with various types of injuries were more than 84 points (Table 3).

**Table 3:** Post-treatment assessment of 61 Patients with Fifth Metatarsal Base Fracture

Injury type	Fracture healing time (weeks)	AOFAS rating				Related complications
		Excellent	Good	Fair	Poor	
Without LCAL injury	8.2 ± 0.6	19	3	0	0	None
Partial LCAL injury (Grade I, II injuries)	8.5 ± 1.4	19	5	0	0	None
Grade III injury	8.3 ± 0.9	5	1	0	0	None
With avulsion fracture	8.6 ± 1.2	7	2	0	0	None
*p* value	0.701^a^ 0.673^b^ 0.09^c^ 0.983^d^ 0.19^e^ 0.27^f^	0.702^a^ 1^b^ 0.613^c^ 1^d^ 1^e^ 1^f^				

LCAL, lateral collateral ankle ligament. Grade I injury, partial tear of a ligament. Grade II injury, incomplete tear of a ligament, with moderate functional impairment. Grade III injury, complete tear and loss of integrity of a ligament. AOFAS, American Orthopaedic Foot and Ankle Society

^a^*p* value between Without LCAL injury and Partial LCAL injury

^b^*p* value between Without LCAL injury and Grade III injury

^c^*p* value between Without LCAL injury and with avulsion fracture

^d^*p* value between Partial LCAL injury and Grade III injury

^e^*p* value between Partial LCAL injury and with avulsion fracture

^f^*p* value between Grade III injury and with avulsion fracture

## Discussion

### Epidemiological characteristics of patients with fifth MBF combined with LCAL injury

fifth MBF is one of the most common foot injuries. Kane et al. noted that zone I fracture accounts for 51% of fifth metatarsal base fractures [4]. However, so far, no study has reported on the incidence of fifth MBF combined with LCAL injury. According to this study, the incidence of fifth MBF combined with LCAL injury accounted for 63.93% of all fifth metatarsal base fracture, with an average age of 43 (range, 20–69) years. Moreover, there was no significant difference between the patients without LCAL injury and the patients with LCAL injury in terms of age (*p* = 0.67, Table 1) and gender (*p* = 0.575, Table 1).

As for ankle sprains, ATFL is the most vulnerable ligament in the ankle, followed by CFL and PTFL [13, 14]. Evulsion fracture after an ankle sprain generally indicates that at least one LCAL is injured [15, 16], while a fifth MBF with avulsion fracture suggests an LCAL injury. In this study, we found no significant difference in age and gender between partial ligament injury (Grade I and II injuries), complete ligament rupture (Grade III injury), and avulsion fracture (lateral malleolus and talus). In terms of incidence, fifth MBF with Grade I and II LCAL injuries was the most common (61.54%, Table 2), followed by avulsion fracture with an incidence of 23.08% (Table 2), and finally Grade III ligament injury with an incidence of 15.38% (Table 2).

### Injury mechanism of fifth MBF combined with LCAL injury

The injury mechanism of the fifth metatarsal base avulsion fracture mainly caused by forces by the peroneus brevis tendon or the lateral band of the plantar fascia during foot inversion [17]. Currently, it still remains controversial, which are the main injured tendons. DeVries et al. conducted cadaveric experiments and discovered that the fracture in the proximal area A of the fifth metatarsal was caused by the lateral band of the plantar aponeurosis, while the fracture in the area B and C was caused by the short peroneal [18]. Moreover, Richli et al. attributed the fifth MBF to the lateral band of the plantar aponeurosis [19]. Yet, the specific mechanism leading to fifth MBF and LCAL injury is still unclear, which may be related to the ankle and foot position at the time of injury, the anatomical characteristics at the ligament attachment point, and the degree of ligament tension [18].

### Treatment of the fifth MBF combined with LCAL injury

According to Lawrence-Botte classification, fractures of the proximal fifth metatarsal are divided into zone I (base avulsion fracture), zone II (Jones' fracture), and zone III (proximal diaphyseal fracture) [5]. For fifth MBF without displacement, good therapeutic results can be achieved by conservative treatment [20]. The accepted treatment method for the fifth MBF with displacement more than 2 mm and involving more than 30% of the fifth metatarsal base-cuboid joint is operative intervention [21, 22]. In this study, an LCAL injury needed to be actively confirmed before surgery. In case of an LCAL injury confirmed, the following strategy was applied: 1. ligament exploration during fracture surgery was suggested to be made in the case of the following circumstances: (1) a complete ligament rupture was confirmed before the surgery, or if the anterior drawer test and inversion test were positive after anesthesia; (2) there was an avulsion fracture of parts such as lateral malleolus and talus; (3) the patient was young, with high demand for sports. 2. Fixation was suggested to be made after the surgery of LCAL injury, and functional exercise was simultaneously delayed. After this, postoperative treatment plan was applied, the average follow-up time was 16.8 months, and no obvious related complications such as lateral ankle instability, pain, and nonunion of fracture were found.

This study has a few limitations. This is a retrospective study with small sample size (power 0.87). In addition, a specific mechanism leading to fifth MBF and LCAL injury was not examined.

To sum up, in this study, we discovered a relatively high incidence of fifth MBF combined with LCAL injury. The confirmation of an LCAL injury is important for formulating treatment plans, and postoperative functional exercises. No additional ligament surgery exploration is recommended for fifth MBF with Grade I and II LCAL injuries; however, additional ligament exploration is recommended for fifth MBF with Grade III LCAL injury or avulsion fracture.

## Data Availability

Not applicable.

## Abbreviations

fifth MBF: Fifth metatarsal base fracture
LCAL: Lateral collateral ankle ligament
ATFL: Anterior talofibular ligament
CFL: Calcaneofibular ligament
PTFL: Posterior talofibular ligament
AOFAS: American Orthopaedic Foot and Ankle Society
AP: Anteroposterior

## References

[1] Petrisor BA, Ekrol I, Court-Brown C (2006). The epidemiology of metatarsal fractures. Foot Ankle Int.

[2] Cakir H, Van Vliet-Koppert ST, Van Lieshout EM, De Vries MR, Van Der Elst M, Schepers T (2011). Demographics and outcome of metatarsal fractures. Arch Orthop Trauma Surg.

[3] Hasselman CT, Vogt MT, Stone KL, Cauley JA, Conti SF. Foot and ankle fractures in elderly white women. Incidence and risk factors. J Bone Jt Surg Am. 2003;85(5):820–4. 10.2106/00004623-200305000-00008. 10.2106/00004623-200305000-00008 12728031

[4] Kane JM, Sandrowski K, Saffel H, Albanese A, Raikin SM, Pedowitz DI (2015). The epidemiology of fifth metatarsal fracture. Foot Ankle Spec.

[5] Lawrence SJ, Botte MJ (1993). Jones' fractures and related fractures of the proximal fifth metatarsal. Foot Ankle.

[6] Strayer SM, Reece SG, Petrizzi MJ (1999). Fractures of the proximal fifth metatarsal. Am Fam Phys.

[7] Hertel J (2002). Functional anatomy, pathomechanics, and pathophysiology of lateral ankle instability. J Athl Train.

[8] Krips R, de Vries J, van Dijk CN. Ankle instability. Foot Ankle Clin. 2006;11(2):311–29. 10.1016/j.fcl.2006.02.003. 10.1016/j.fcl.2006.02.003 16798514

[9] Kobayashi T, Gamada K. Lateral Ankle Sprain and Chronic Ankle Instability: A Critical Review. Foot Ankle Spec. 7. United States: © 2014 The Author(s). 2014. p. 298–326. 10.1177/1938640014539813 24962695

[10] Chorley JN, Hergenroeder AC (1997). Management of ankle sprains. Pediatr Ann.

[11] Hollis JM, Blasier RD, Flahiff CM. Simulated lateral ankle ligamentous injury. Change in ankle stability. Am J Sports Med. 1995;23(6):672–7. 10.1177/036354659502300606. 10.1177/036354659502300606 8600732

[12] Kitaoka HB, Alexander IJ, Adelaar RS, Nunley JA, Myerson MS, Sanders M (1994). Clinical rating systems for the ankle-hindfoot, midfoot, hallux, and lesser toes. Foot Ankle Int.

[13] Ferran NA, Maffulli N (2006). Epidemiology of sprains of the lateral ankle ligament complex. Foot Ankle Clin.

[14] Hølmer P, Søndergaard L, Konradsen L, Nielsen PT, Jørgensen LN (1994). Epidemiology of sprains in the lateral ankle and foot. Foot Ankle Int.

[15] Brostroem L. Sprained ankles. I. Anatomic lesions in recent sprains. Acta Chir Scand. 1964;128:483–95. 14227127

[16] Haraguchi N, Kato F, Hayashi H (1998). New radiographic projections for avulsion fractures of the lateral malleolus. J Bone Jt Surg Br.

[17] Cheung CN, Lui TH (2016). Proximal fifth metatarsal fractures: anatomy, classification, treatment and complications. Arch Trauma Res.

[18] DeVries JG, Taefi E, Bussewitz BW, Hyer CF, Lee TH (2015). The fifth metatarsal base: anatomic evaluation regarding fracture mechanism and treatment algorithms. J Foot Ankle Surg.

[19] Richli WR, Rosenthal DI (1984). Avulsion fracture of the fifth metatarsal: experimental study of pathomechanics. AJR Am J Roentgenol.

[20] Bica D, Sprouse RA, Armen J (2016). Diagnosis and management of common foot fractures. Am Fam Phys.

[21] Wu GB, Li B, Yang YF (2018). Comparative study of surgical and conservative treatments for fifth metatarsal base avulsion fractures (type I) in young adults or athletes. J Orthop Surg (Hong Kong).

[22] Valkier C, Fallat LM, Jarski R (2020). Conservative versus surgical management of fifth metatarsal avulsion fractures. J Foot Ankle Surg.

